# Supplementary material for: The varied sources of faculae-forming brines in Ceres’ Occator crater emplaced via hydrothermal brine effusion
Source: Nat Commun. 2020 Aug 10;11:3680. doi: 10.1038/s41467-020-15973-8 (PMC7417532; doi:10.1038/s41467-020-15973-8)
Supplement: Supplementary file 1 — Supplementary Information [file 41467_2020_15973_MOESM1_ESM.pdf]

Supplementary Information for:

The Varied Sources of Faculae-Forming Brines in Ceres' Occator Crater Emplaced via Hydrothermal Brine Effusion

Scully et al.

J. E. C. Scully(1*), P. M. Schenk(2), J. C. Castillo-Rogez(1), D. L. Buczkowski(3), D. A. Williams(4), J. H. Pasckert(5), K. D. Duarte(6), V. N. Romero(6), L. C. Quick(7), M. M. Sori(8), M. E. Landis(9), C. A. Raymond(1), A. Neesemann(10), B. E. Schmidt(6), H. G. Sizemore(11), C. T. Russell(12).

(1)Jet Propulsion Laboratory, California Institute of Technology, Pasadena, California, USA,
(2)Lunar and Planetary Institute, Houston, Texas, USA,
(3)Johns Hopkins University Applied Physics Laboratory, Laurel, MD, USA,
(4)School of Earth and Space Exploration, Arizona State University, Tempe, Arizona, USA,
(5)Institute für Planetologie, University of Münster, Münster, Germany,
(6)Georgia Institute of Technology, Atlanta, Georgia, USA,
(7)NASA Goddard Space Flight Center, Greenbelt, Maryland, USA,
(8)Lunar and Planetary Laboratory, Tucson, Arizona, USA,
(9)Laboratory for Atmospheric and Space Physics, University of Colorado Boulder, Boulder, Colorado, USA,
(10)Free University of Berlin, 14195 Berlin, Germany,
(11)Planetary Science Institute, Tucson, Arizona, USA,
(12)University of California, Los Angeles, California, USA.

*Corresponding Author: Dr. J. E. C. Scully: (Jennifer.E.Scully@jpl.nasa.gov)

Supplementary Discussion

Description of Map Units and Correlation of Map Units

Crater floor material with lobate material mantling (cflm)

Description:

This unit is located in the northwestern interior of the crater. The mantled crater floor material has a broad-scale irregular/blocky surface texture, which frequently grades to a smoother appearance locally (Supplementary Figure 6a). It shares certain contacts with the mantled terrace and massif materials. However, there are no superposition relations between these three units. The mantled crater floor material shares certain and approximate contacts with the three types of lobate materials, all of which superpose the crater floor material. This unit is also superposed by the talus material (with which it shares certain contacts) and the discontinuous bright material (with which it shares approximate contacts) (Supplementary Figure 12). This unit has an intermediate brightness, and as such we mapped it using a standard deviation stretch of n=2.5 on the basemap.

Interpretation:

We interpret this unit as the original floor material of the crater. The XM2 data reveals that the majority of this unit is coated to varying extents by a comparatively smoother veneer of lobate material, which we interpret was emplaced as the lobate material slurry flowed around the crater interior prior to solidification. The margins of the lobate material coating are rarely discernible; when the margins are observable we map a separate unit of lobate material (Supplementary Figure 6a).

Terrace material with lobate material mantling: thick (tlmtk) and thin (tlmtn)

Description:

The mantled terrace material is composed of steps, or terraces, which are roughly concentric to the crater rim. The morphology of these steps is more clearly expressed, and the surface texture rougher, in the terrace material with thin lobate material mantling and is more muted, with a comparatively smoother surface texture, in the terrace material with thick lobate material mantling (Supplementary Figure 6b). This unit forms an almost continuous ring around the interior of the crater. On the lower slopes of the crater wall, there are instances where the mantled terraces contain fractures that are parallel to the crater rim (Supplementary Figure 6c). Fractures occur throughout Occator: we map those with a fresher

appearance as sharp grooves and those with a more degraded appearance as subdued grooves. The unit shares certain contacts with the mantled crater floor material and we do not observe superposition relations between the units. The mantled terrace material is superposed by all three types of lobate materials, with which it shares certain and approximate contacts. This unit is also superposed by the talus material, with which it shares certain contacts (Supplementary Figure 12). This unit has an intermediate brightness, and as such we mapped it using a standard deviation stretch of n=2.5 on the basemap.

Interpretation:

We interpret that this unit formed via relatively coherent crater-wall collapse during/shortly after the crater-forming impact[1]. The XM2 data provides additional information about the formation of this unit: it is coated to varying extents (i.e., thick versus thin mantling) by a comparatively smoother veneer of lobate material, which likely formed as the lobate material slurry flowed around the crater interior prior to solidification. The coating of lobate material rarely has discernible margins; when the margins are observable we map a separate unit of lobate material (Supplementary Figure 6b). We interpret that the fractures parallel to the crater rim formed when the lobate material that mantles the terraces splashed up part of the crater wall and gradually creeped back down as it solidified.

Massif material (m)

Description:

The massif material is adjacent to the central pit on the western, eastern and northern sides. The massif material displays a more blocky surface texture than the mantled crater floor material, and is at a distinctly higher elevation, as indicated by the basal scarp linear feature on the geologic map (Figure 1a). The boundary of the massif material does not always align with the basal scarp because the definition of the massif material is also based on its surface texture, and not on topography alone, like the basal scarp. The unit shares certain contacts with the mantled crater floor material and talus material: there is no clear superposition relation between the unit and the mantled crater floor material, while the talus material superposes the massif material. The massif material is also superposed by the interspersed lobate material, hummocky lobate material and discontinuous bright material, with which it shares certain contacts (Supplementary Figure 12). This unit has an intermediate brightness, and as such we mapped it using a standard deviation stretch of n=2.5 on the basemap.

Interpretation:

We interpret this unit as a higher standing region of the original crater floor than the mantled crater floor material, which resulted in the lobate material slurry flowing around the massif material, instead of coating it. The massif material may be the solid remnants of an early and short-lived liquid-water-dominated central peak, which mostly drained away into impact-induced fractures to form the central pit[2].

Lobate material: smooth (ls), interspersed (li) and hummocky (lh)

Description:

In addition to mantling the crater floor material and terrace material, the lobate material forms a large, thick sheet in the southern and eastern crater interior, and isolated pond-like deposits throughout the rest of the crater floor and the terraces. We divide the lobate material into three sub-units based on the surface texture: smooth, interspersed and hummocky (Supplementary Figure 8a-c). The endmember sub-units have smooth and hummocky surface textures, and are consistent with pre-XM2 observations. The XM2 data facilitates the definition of an intermediate sub-unit: smooth lobate material that is interspersed with knobs (mapped as domes or mounds) and striations (mapped as lineaments), which is referred to as interspersed lobate material. The detailed texture of this unit was not clearly visible in the pre-XM2 data.

When there are clearly defined flow margins between the sub-units, it is possible to place a certain contact between them. However, these three sub-units often grade into one another, which is illustrated by approximate contacts on the geologic map. Moreover, sometimes the end of a smooth lobate material flow displays the texture of the interspersed lobate material (i.e. it is striated), without a contact separating the two. In these cases we map the unit as smooth lobate material, and use the lineament linear feature to indicate the presence of striations (Supplementary Figure 8d). The flows of lobate material often superpose one another. For example, in the northeastern crater interior, a pond-like deposit of smooth lobate material (flow #1) is superposed to the south by the high-standing hummocky lobate material (flow #2), and to the north by a multi-lobed flow of interspersed lobate material (flow #3) (Supplementary Figure 5b). The interspersed lobate material (flow #3) originates from a ledge at the base of the crater wall, and then flows toward the crater interior under the control of the local topography. The flow of interspersed lobate material (flow #3) is in turn

superposed by a topographically-controlled flow of smooth lobate material (flow #4), which contains striations at the end of the flow.

All occurrences of the lobate material superpose the margins of the mantled crater floor material, mantled terrace material and massif material. These units share certain contacts with the lobate material where there is a clearly observable boundary and approximate contacts when the boundary is less well defined. The lobate material is superposed by the talus material and spur material (with accurate contacts) and by the discontinuous bright material (with approximate contacts because of the diffuse nature of the discontinuous bright material) (Supplementary Figure 12).

We map the knobs as domes or mounds in our geologic map: the domes tend to be larger (average diameters in the high hundreds of meters) and more isolated while the mounds tend to be smaller (average diameters in the low hundreds of meters) and more clustered. While the majority of the mounds do not have a distinct morphology (unclassified), there are mounds with distinctly conical shapes, and those with tops that are flat, blocky or contain a depression (Supplementary Figure 7a). To avoid cluttering the main geologic map with the ~450 individual mounds, we represent clusters of mounds by using a surface feature symbol where 5 or more mounds are located in close proximity to one another. We identify ring-mold craters in the lobate material and in the adjacent mantled terrace material.

The lobate material has an intermediate brightness, and as such we mapped it using a standard deviation stretch of n=3 - 5 on the basemap. We used a slightly higher value than the previous units that have intermediate brightness (n=2.5) in order to enhance the surface texture of the lobate materials.

Interpretation:

We interpret that the lobate material was emplaced as a slurry of impact-melted water, salts in solution and blocks of unmelted silicates and salts flowed around the crater interior shortly after the crater's formation[3]. The sub-units of lobate material that grade into one another were likely emplaced at the same/similar times, while those with more clearly defined flow margins were separated by longer timescales. The striations in the interspersed lobate material formed as the material underwent the final stages of flow prior to solidification. The smooth lobate material lacks the processes/features that formed the striations and knobs in the interspersed lobate material. The later injection of a water/salt intrusion is proposed to have inflated the hummocky lobate material, resulting in its distinctive texture[4]. There are three possible formation mechanisms for the domes and

mounds: (a) pinnacles around which the lobate material flowed, (b) blocks of unmelted silicates and salts that were entrained in the lobate material slurry, and (c) eruptive and/or frost-heave like processes derived from the solidification and expansion of the water-ice-rich lobate material[5]. Water-ice-rich material is proposed to be involved in the formation of the ring-mold craters in Occator[6], consistent with the proposition that water ice is present in the lobate material.

Bright material: continuous (bc), moderately discontinuous (bdm) and discontinuous (bd)
Description:

The bright material units correspond to the Cerealia Facula (continuous and discontinuous bright material), Pasola Facula (continuous bright material only) and Vinalia Faculae (continuous, moderately discontinuous and discontinuous bright material). Cerealia Facula is located in the center of the crater, in and surrounding the central pit. Pasola Facula is located on a ledge that is part of the massif to the west of the central pit. Vinalia Faculae are within the hummocky lobate material in the eastern crater floor (Figure 1a). While Cerealia Facula and Pasola Facula are generally brighter than Vinalia Faculae, the texture of the continuous material between all three faculae is consistent. The texture of the discontinuous bright material is also consistent between Cerealia and Vinalia Faculae: it is more diffuse than the continuous bright material (Supplementary Figure 1). The moderately discontinuous bright material has an intermediate texture between the continuous and discontinuous bright materials: it is more diffuse than the continuous bright material but more continuous than the discontinuous bright material (Supplementary Figure 1). Thus, we use the distinct textural appearances of the material in the XM2 data as the main discriminator between the bright material sub-units, rather than their brightnesses.

The continuous and moderately discontinuous bright materials are often roughly circular deposits surrounded by the discontinuous bright material. The discontinuous bright material is in turn surrounded by even more diffusely dispersed points of bright material, which we represent on the map by use of the faint mottled bright material surface feature. The continuous and moderately discontinuous bright materials have clearly defined margins that result in these sub-units having certain contacts with the discontinuous bright material, the talus material and the dark material. There are instances where the talus and dark material cross-cut the continuous and moderately discontinuous bright materials; elsewhere the superposition relations are less clear. The discontinuous bright material superposes the

mantled crater floor material, the interspersed lobate material and the hummocky lobate material. It shares approximate contacts with each of these units because the diffuse texture of the sub-unit means a definitive edge is not present. The discontinuous bright material shares certain contacts with the talus material, dark material and massif material. The discontinuous bright material often superposes the massif material, while the talus and dark material often superpose the discontinuous bright material. The diffuse nature of the discontinuous bright material means there are no clearly discernible superposition relations between it and the other bright materials (Supplementary Figure 12). Cerealia Tholus (the central dome) is located entirely within the continuous bright material of Cerealia Facula.

All faculae appear over-saturated in the previously used standard deviation stretches of the basemap ($n \leqq 5$). Thus, in order to make features within the faculae clearly visible, we used a standard deviation stretch of n=15 while mapping the Cerealia Facula and Pasola Facula and of n=7 while mapping the Vinalia Faculae.

Interpretation:

Our observations of the faculae are consistent with them being the remnants of brines sourced in the subsurface that lost their liquid water component on Ceres' surface[3,7-8]. If the entire Cerealia Tholus is composed of continuous bright material, rather than being coated by it, ~2 km^3 of bright material is required. We calculated this volume by approximating the shape of the dome as a partial hemisphere with a radius of ~1 km and a height of ~700 m (radius and height based on ref. 2). We further discuss the insights into faculae formation derived from our geologic mapping throughout the main text.

Dark material (d)

Description:

The units of dark material are patches of distinctly low brightness material that are located within the continuous and discontinuous bright materials. There are distinct margins between the dark material and the bright material, which we map with certain contacts. The shapes of the dark material patches are irregular, and some are located around a central depression (Figures 1-2). The dark material sometimes superposes the bright material, and in other instances the bright material superposes the dark material (Figure 2a). A standard deviation stretch of n=5 on the basemap allowed us to accurately map the contact between the dark and bright material.

Interpretation:

We interpret that the areas of dark material surrounding a central depression are dark ejecta that was excavated by an impact cratering event, which cut through the bright material to the underlying darker material. Other areas of dark material may also be dark ejecta, with a central impact crater that is smaller than the resolution limit of the basemap. Some areas of dark material are also likely regions that were not covered by bright material or where mass wasting deposited dark material on top of bright. For further discussion of the dark material see the main text.

Talus material (t)

Description:

The talus material is located at steeply sloping regions on the walls of the crater, at the edges of terraces, at scarps in the mantled crater floor material and on the lower slopes of the massifs surrounding the central pit. This unit consists of narrow streaks or broad lobes of material that tend to be finer grained that the resolution limit of the basemap, and which appear to have flowed to a topographically lower point. This unit has a fresh appearance that allows for all of its contacts to be defined as certain. The talus material superposes all of the geologic units in the map, apart from the spur material, with which there is no clearly defined superposition relation. There are complex superposition relations between the talus material and the bright material. Sometimes the talus clearly superposes the bright material, sometimes the bright material superposes the talus and in other locations the superposition relations are less clear because of the high contrast between the brightnesses of the materials (Supplementary Figure 12). While some streaks/lobes within the talus material are almost as bright as the faculae, the majority of the unit is of intermediate-dark brightness. Thus, we mapped this unit using a standard deviation stretch of n=2 on the basemap.

Interpretation:

We interpret the talus material as dry mass wasting deposits. Their fresh appearance indicates that they were relatively recently deposited. The majority of talus deposits occur on steep slopes, which is consistent with the mass wasting being triggered by over-steepening and/or seismic shaking from the formation of nearby impact craters. Note that the pit chains that cross-cut the faculae are coated by dark talus, but we map them as linear features (sharp or subdued grooves in the geologic map), instead of geologic units of dark talus material, to avoid cluttering the map with many closely spaced geologic units.

Spur material (s)

Description:

This unit consists of blocky outcrops that are most often located upslope of the talus material. The spur material often occurs at a break in slope, for example, at the crater rim or at the crest of a terrace. This unit has a fresh appearance that allows for all of its contacts to be defined as certain. The spur material superposes the mantled terrace material and the massif material. There are no clearly defined superposition relations between the spur and talus materials (Supplementary Figure 12). The spur material has an intermediate-dark brightness. Thus, we mapped this unit using a standard deviation stretch of n=2 on the basemap.

Interpretation:

We interpret the spur material as the source outcrops for the dry mass wasting deposits. Their fresh appearance indicates that they are relatively recent.

Supplementary Methods

Cliff stress model

There is widespread talus material around the crater wall, which formed throughout Occator's history as granular material eroded from the crater rim and cascaded down the wall. The XM2 data reveals that upslope of the talus material, there are often occurrences of unusually steep topography (called cliffs hereafter) along crater rims (Supplementary Figure 13). Landslides downslope of the cliffs may represent failed cliffs that broke off from the crater rim when subjected to sufficiently high stresses.

We model the state of stress expected in the cliffs by using beam theory to calculate the bending and shear stresses throughout an overhanging cliff on Ceres under a variety of assumed geometries. Cliff shape cannot be uniquely determined from the current topographic data. However, we set an upper constraint on the plausible stresses a cliff could be subjected to, by considering an end-member case where the cliff is overhanging. These overhanging models are end-member cases that represent the maximum stresses the cliffs could obtain; in reality, cliffs may simply be steep rather than overhanging, and have less stress. We use analytical equationsthat have been shown to accurately calculate stresses, with results comparable to finite element modelling[9]. The maximum bending and shear stresses are compared to the tensile and shear strength of the assumed material; failure occurs when the

stresses exceed the strength. Compressive strength is not considered, as tensile failure always occurs more readily in these cases. Gravitational acceleration is set to 0.27 m s^{-2} in all cases.

Geometries are uncertain but are based on XM2 images. For example, we consider an 800-m-long overhang that is 100-m-thick, jutting out over empty space, and an 800-m-long overhang that is 200-m-thick at the overhang-rim interface but narrows to a point at the end of the overhang. These geometries may represent the cliff seen in Supplementary Figure 13. Bending and shear stress are maximized at the interface between the overhang and the crater rim. If the overhang is composed of H_2O ice (assumed density of 920 kg m^{-3}), we calculate maximum shear stresses to be >14 MPa in both cases, resulting in failure because ice shear strength is a few MPa. This failure is robust with respect to the assumed thickness and shape of the cliff if the overhang is 800-m-long, and thinner cliffs may additionally fail from bending stress exceeding tensile strength. However, the average Cerean crust (assumed density of 1287 kg m^{-3}) contains silicates and may contain methane clathrate hydrate and/or salts[10-11]. The presence of methane clathrate hydrate, in particular, could make the average crust much stronger[12], allowing even the end-member case of an overhanging cliff to have stresses (a few 10s of MPa for shear stress in the most extreme cases) less than the strength of the material. Therefore, the average Cerean crust could form cliffs that adequately resist the stress of their own weight to prevent failure under normal conditions.

We find that while a purely water ice overhang would likely fail from shear stress, cliffs that are composed of average Cerean crustal material (a mixture of silicates, water ice, salt, and clathrates[10,13] have sufficient strength to resist failure from both bending stresses and shear stresses, even if they are overhanging. Thus, it is not necessary to invoke lateral heterogeneity in crustal composition to explain why cliffs are present (and presumably stable) in some locations, and collapsed in others. The persistence of cliffs in certain locations may instead be controlled by thermal factors and/or by the location of impacts, because seismic activity caused by nearby impacts may cause cliff failure.

References

1. Scully, J. E. C., et al. Ceres' Occator crater and its faculae explored through geologic mapping. *Icarus* **320**, 7-23 (2019).
2. Schenk, P. M., et al. The central pit and dome at Cerealia Facula bright deposit and floor deposits in Occator crater, Ceres: morphology, comparisons and formation. *Icarus* **320**, 159-187 (2019).
3. Scully, J. E. C., et al. Synthesis of the special issue: the formation and evolution of Ceres' Occator crater. *Icarus* **320**, 213-225 (2019). And references therein.
4. Buczkowski, D. L., et al. Tectonic analysis of fracturing associated with Occator crater. *Icarus* **320**, 49-59 (2019).
5. Schmidt, B. E., et al. Hydrological evidence of Occator crater: implications from pingo and frost heave morphology. This issue.
6. Krohn, K., et al. Ring-mold craters on Ceres: Evidence for shallow subsurface water ice sources. *Geophys. Res. Lett.* **45**, 8121-8128 (2018).
7. Nathues, A., et al. Sublimation in bright spots on (1) Ceres. *Nature* **528**, 237-240 (2015).
8. De. Sanctis, M. C., et al. Bright carbonate deposits as evidence of aqueous alteration on Ceres. *Nature* **536**, 54-57 (2016).
9. Tokashiki, N. & Aydan, O. The stability assessment of overhanging ryukyu limestone cliffs with an emphasis on the evaluation of tensile strength of rock mass. *Doboku Gakkai Ronbunshuu* **66**, 397–406 (2010).
10. Fu, R. R., et al. The interior structure of Ceres as revealed by surface topography. *Earth Planet. Sci. Lett.* **476**, 153–164 (2017).
11. Ermakov, A. I., et al. Constraints on Ceres' internal structure and evolution from its shape and gravity measured by the Dawn spacecraft. *J. Geophys. Res. Planets* **122,** 2267–2293 (2017).
12. Durham, W. B., et al. The strength and rheology of methane clathrate hydrate. *J. Geophys. Res. Solid Earth,* **108,** B42182 (2003).
13. Russell, C. T., et al. Dawn arrives at Ceres: exploration of a small volatile-rich world. *Science* **353 (6303)**, 1008-1010 (2016).
14. Roatsch, T., et al. Highest resolution mosaic of Ceres derived from Dawn Framing Camera. *American Geophysical Union Fall Meeting*, #P33D-3869 (2018).
15. Pasckert, J. H., et al. Geologic mapping (1:10,000) of Cerealia Facula based on Dawn's high resolution XM2 data. *Lun. Planet. Sci. Conf.*, #2308 (2019).

16. Roatsch, Th., et al. High-resolution Ceres Low Altitude Mapping Orbit atlas derived from Dave Framing Camera images. *Planet. Space Sci.* **140**, 74-79 (2017).
17. Jaumann, R., et al. Topography and geomorphology of the interior of Occator crater on Ceres. *Lun. Planet. Sci. Conf.*, #1440 (2017).
18. Neesemann, A., et al. The various ages of Occator crater, Ceres: results of a comprehensive synthesis approach. *Icarus* **320**, 60-82 (2019).
19. Nathues, A., et al. Recent cryovolcanic activity at Occator crater on Ceres. This issue.
20. Hiesinger, H., et al. Cratering on Ceres: implications for its crust and evolution. *Science* **353 (6303)**, 1003 (2016).

Supplementary Figures

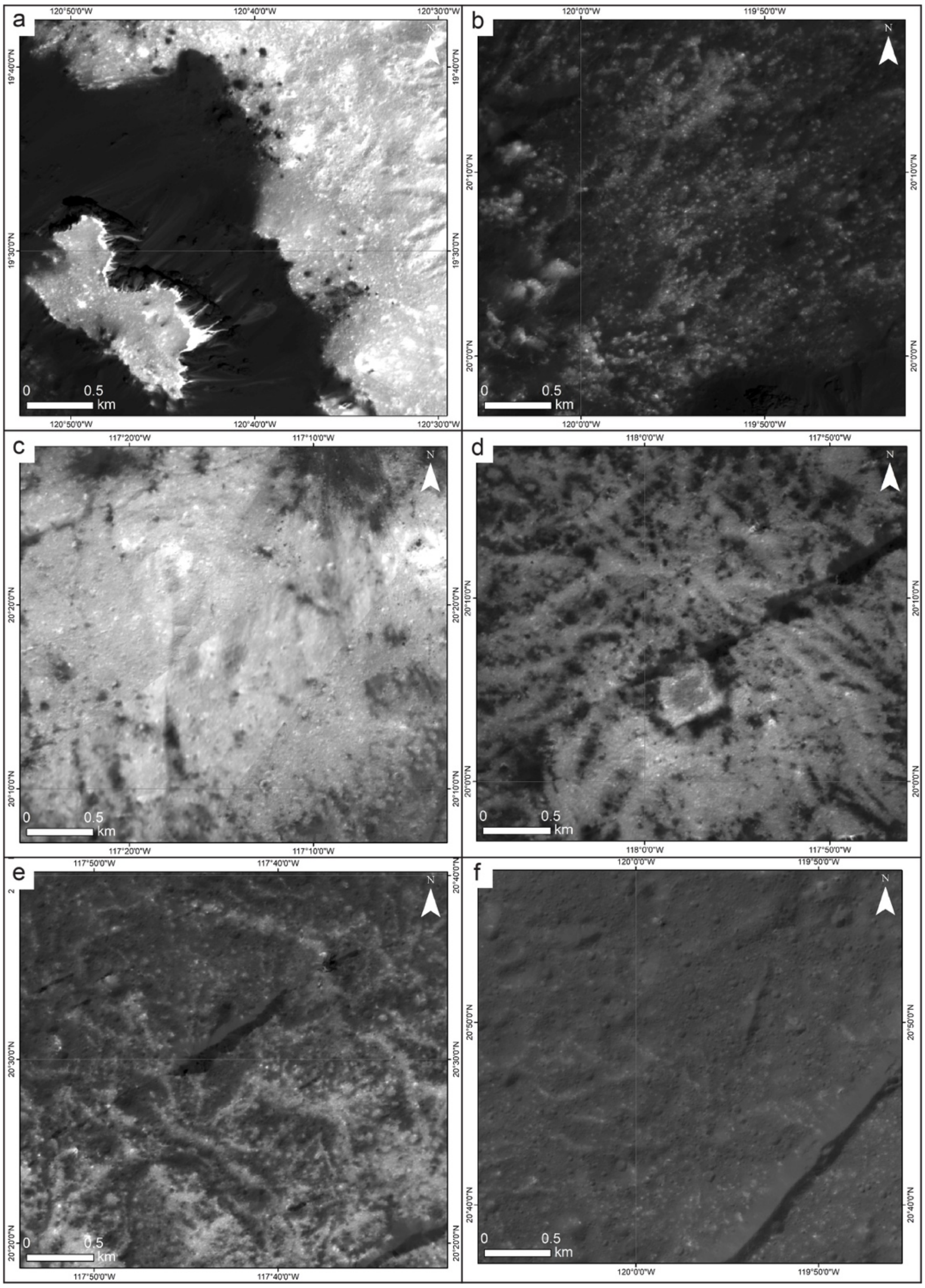

Supplementary Figure 1 Examples of the different types of bright materials. a Continuous bright material of Pasola Facula (left) and Cerealia Facula (right), with dark material in between. **b** Discontinuous bright material of Cerealia Facula. **c** Continuous bright material of Vinalia Faculae. **d** Moderately discontinuous bright material of Vinalia Faculae. **e** Discontinuous bright material of Vinalia Faculae. **f** Faint mottled bright material. All images are taken from the base map: the XM2 clear filter FC mosaic[14].

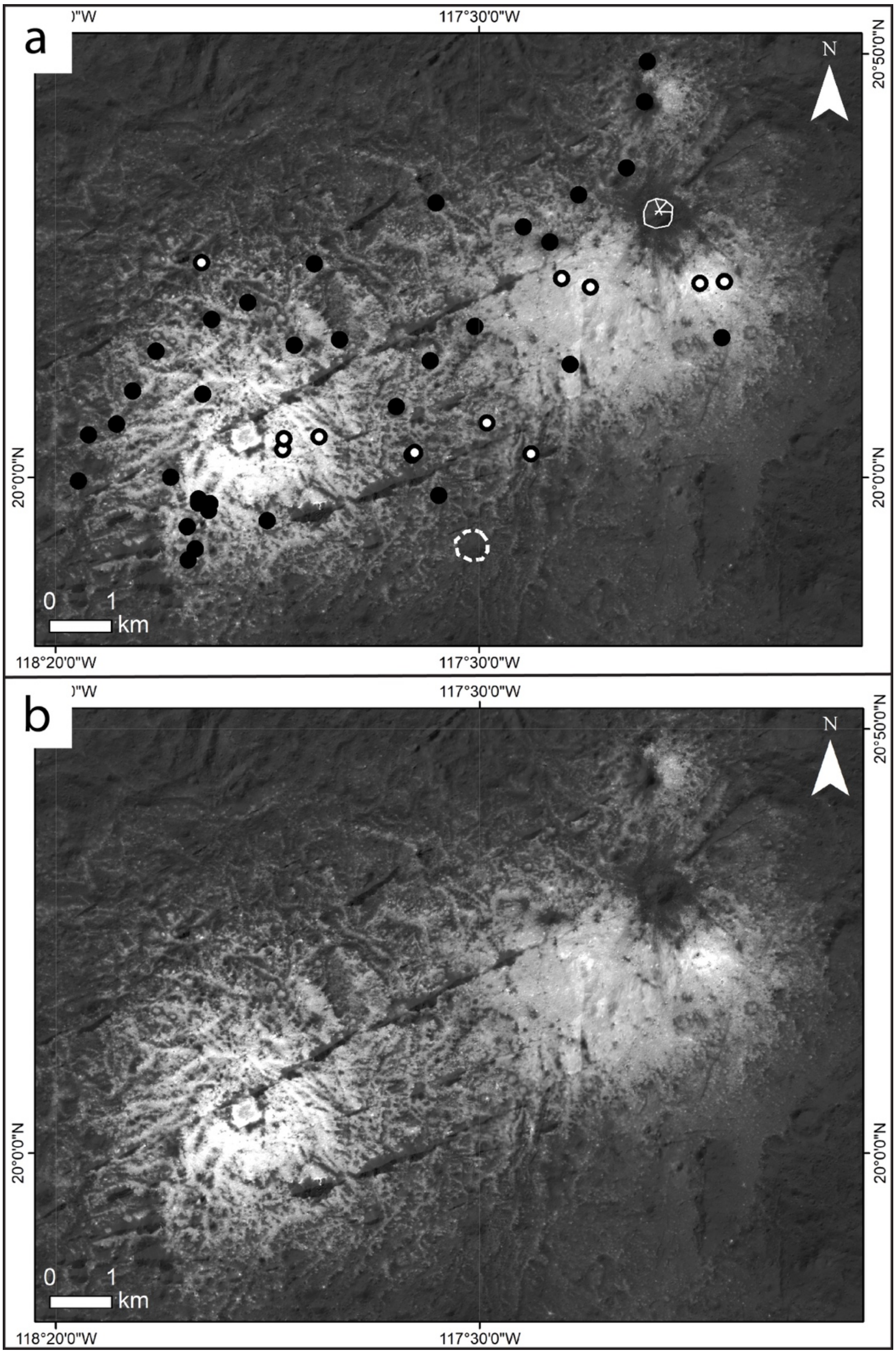

Supplementary Figure 2 Small impact craters near Vinalia Faculae. a A mapped view: white dots indicate craters <400 m in diameter, which excavate bright material, and black dots indicate craters <400 m in diameter, which excavate dark material. The solid white line is a raised rim crater ≧400 m in diameter and the dashed white line is a muted rim crater ≧400 m in diameter. **b** An unmapped view. The small impact craters could have reactivated

the fractures/pit chains that cross-cut Vinalia Faculae. The image is taken from the base map: the XM2 clear filter FC mosaic[14].

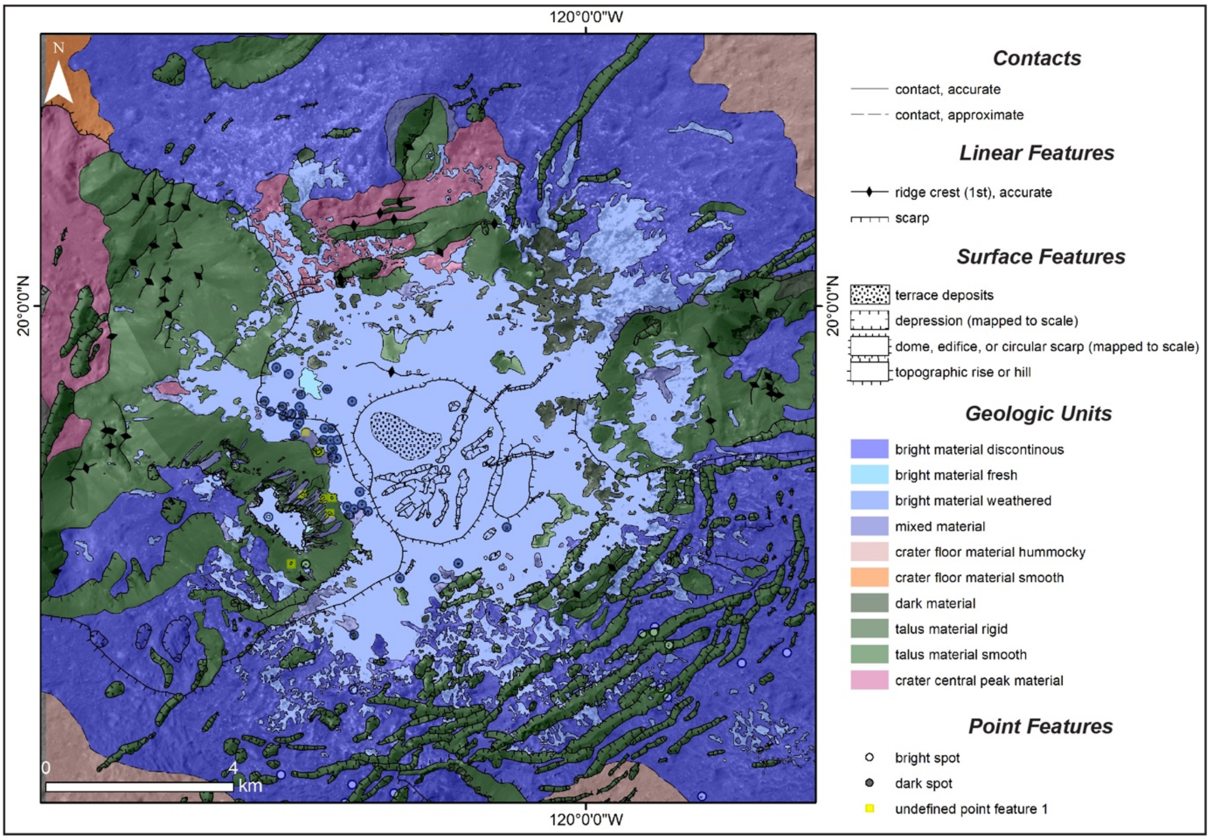

Supplementary Figure 3 Additional geologic map of the Cerealia Facula region. This region was mapped at 1:10,000[15] and incorporated into the main geologic map (Figure 1a). The basemap is shown with no mapping in Supplementary Figure 4.

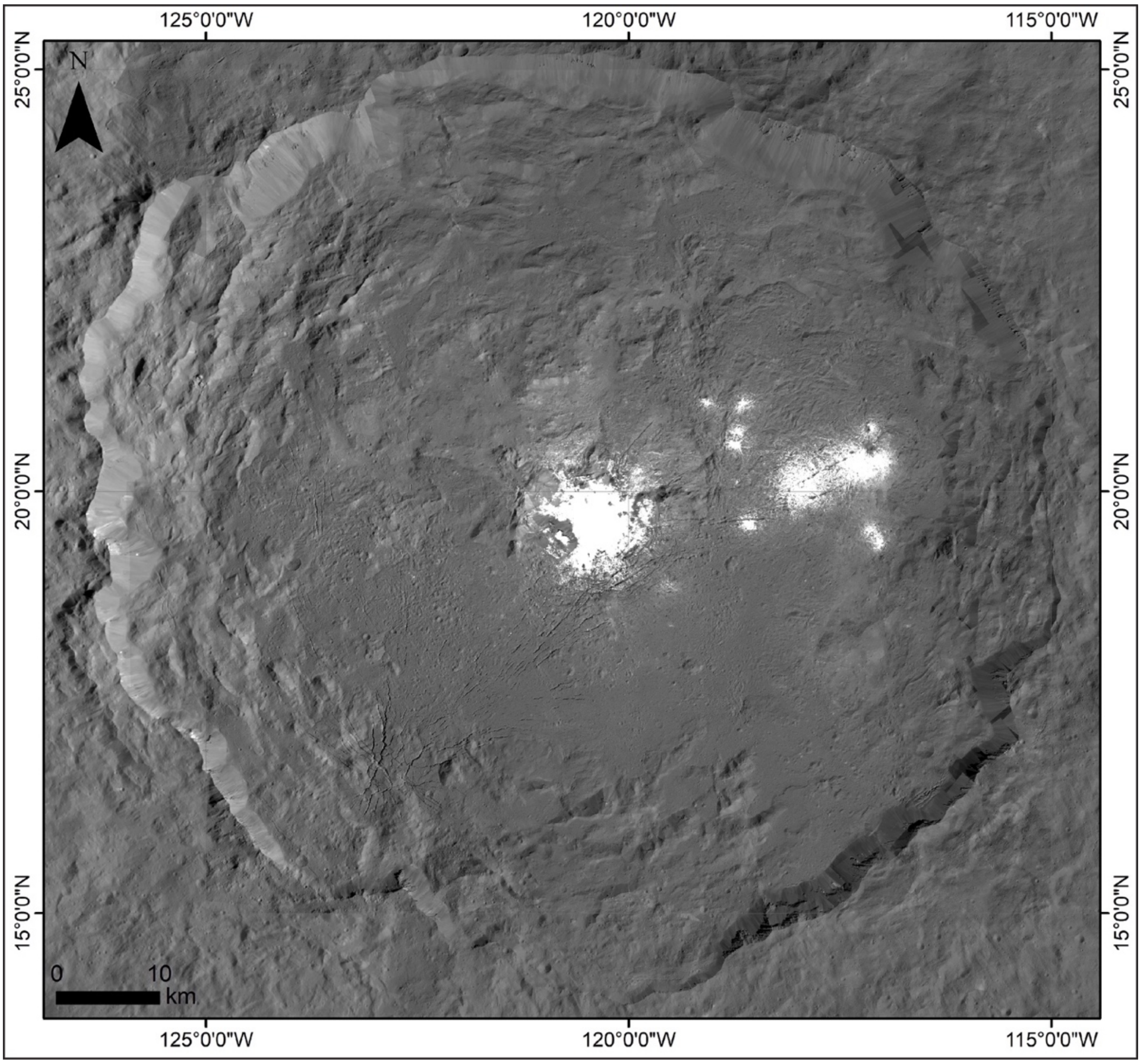

Supplementary Figure 4 The basemap shown with no geologic mapping. The basemap we used is the XM2 clear filter FC mosaic (~3 m/pixel)[14], shown with a standard deviation stretch of n=2.5. The southernmost and westernmost parts of Occator's interior are outside of our basemap. In these areas we supplemented our basemap with a ~10 m/pixel XM2 clear filter FC controlled mosaic and the LAMO clear filter FC controlled mosaic (~35 m/pixel)[16], which is orthorectified onto the LAMO DTM[17].

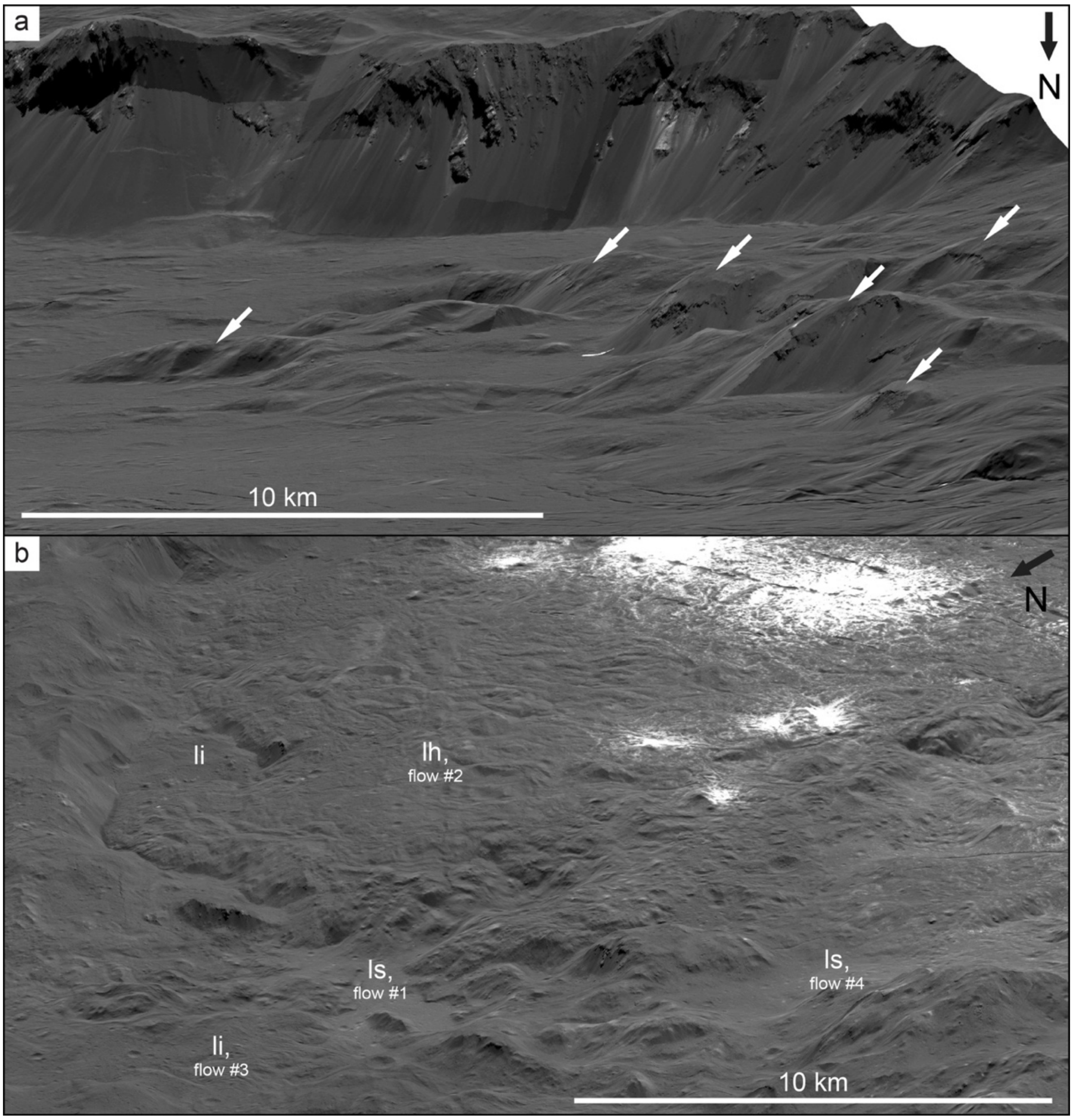

Supplementary Figure 5 Perspective views of lobate material in Occator crater. a Lobate material coating terraces in the southern crater interior, facing the southern crater rim. The cap material is indicated by arrows. There is no vertical exaggeration and to make the perspective view we referenced the base mosaic to the LAMO DTM[17]. **b** Different lobate material flows superposing one another in the northeastern crater interior. The superposition relations between flows #1-4 are detailed in the Supplementary Discussion, subsection Lobate material. The ~10 m/pixel XM2 clear filter mosaic has 2x vertical exaggeration and to make the perspective view we referenced the base mosaic to the LAMO DTM.

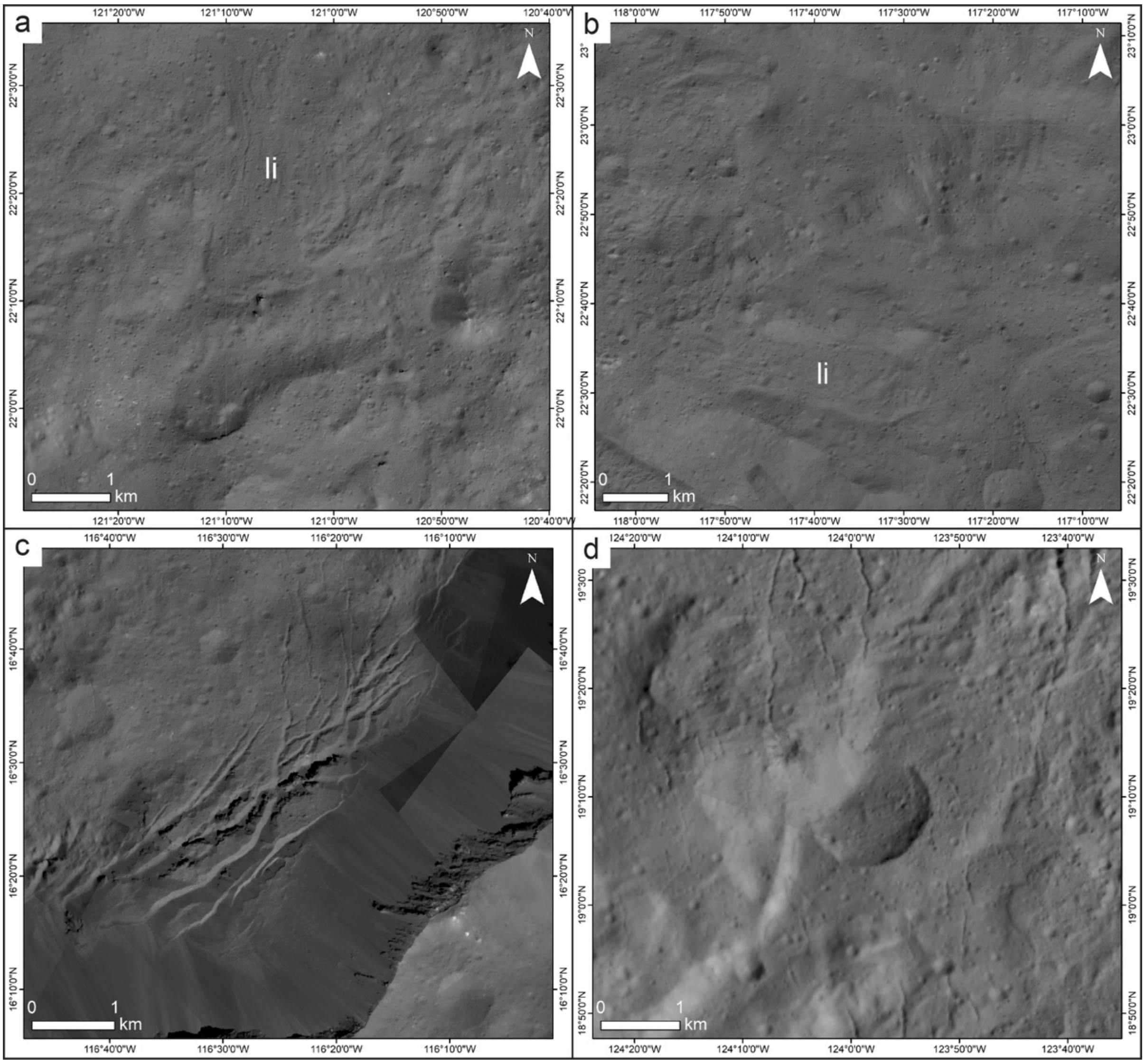

Supplementary Figure 6 Textures/features in the mantled crater floor/mantled terraces. **a** An example of the crater floor material with lobate material mantling fills this image. A distinct flow is labelled as a separate unit of interspersed lobate material (li). **b** An example of the terrace material with thick lobate material mantling is at the top of this image, and the terrace material with thin lobate material mantling is at the bottom of this image. A distinct flow is labelled as a separate unit of interspersed lobate material (li). **c** An example location where the mantled terraces contain fractures that are parallel to the crater rim, which is located in the bottom right corner. **d** A ~500 m thick ridge of lobate material that cross-cuts an impact crater in the mantled terraces. All images are taken from the base map: the XM2 clear filter FC mosaic[14].

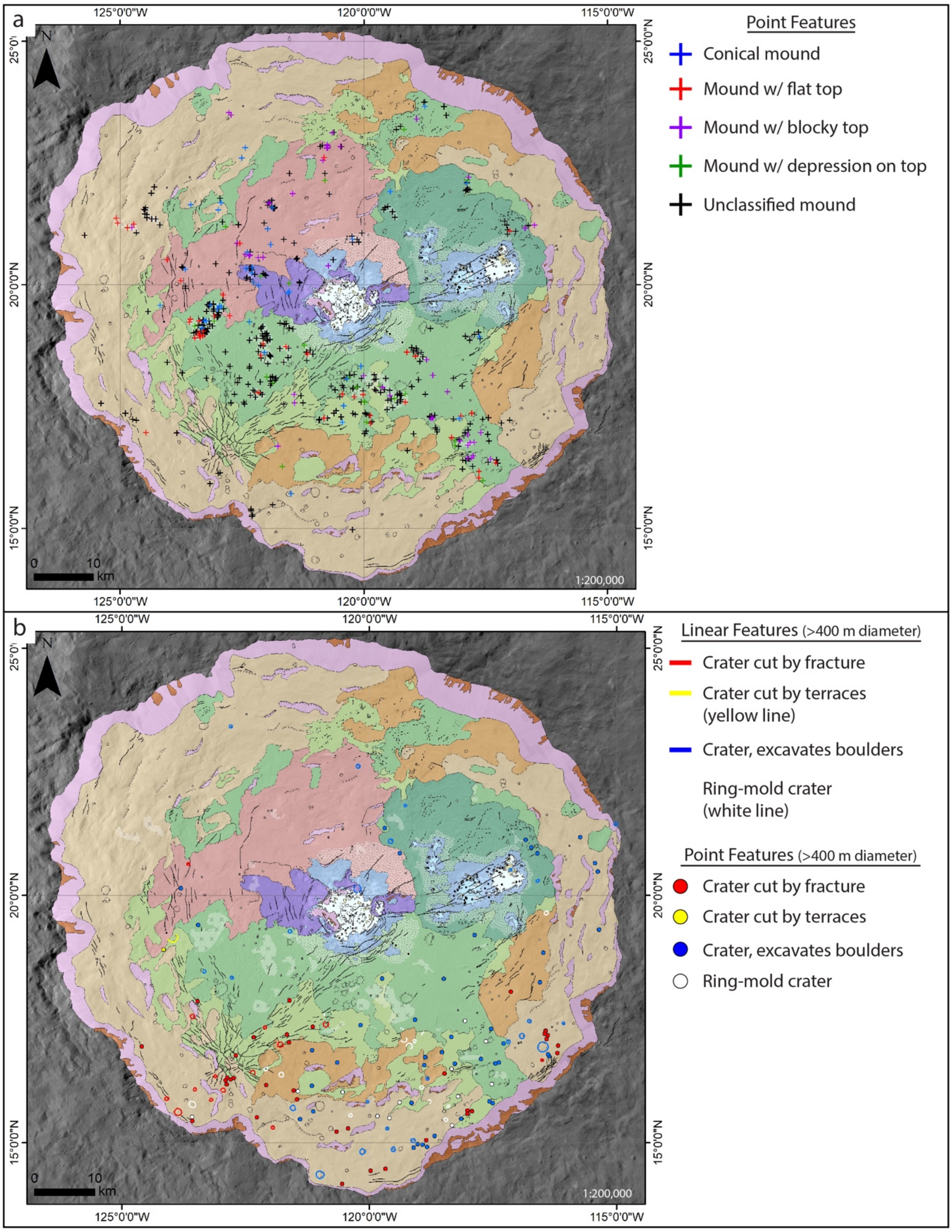

Supplementary Figure 7 XM2-based geologic map with additional features. a Approximately 450 individual mounds, divided into five categories. **b** Craters with distinctive features, separated into linear features (craters with diameters ≧400 m) and point features (craters with diameters <400 m). The legend for the full geologic map is shown in Figure 1a, and the colors and symbols not defined here are the same as in Figure 1a.

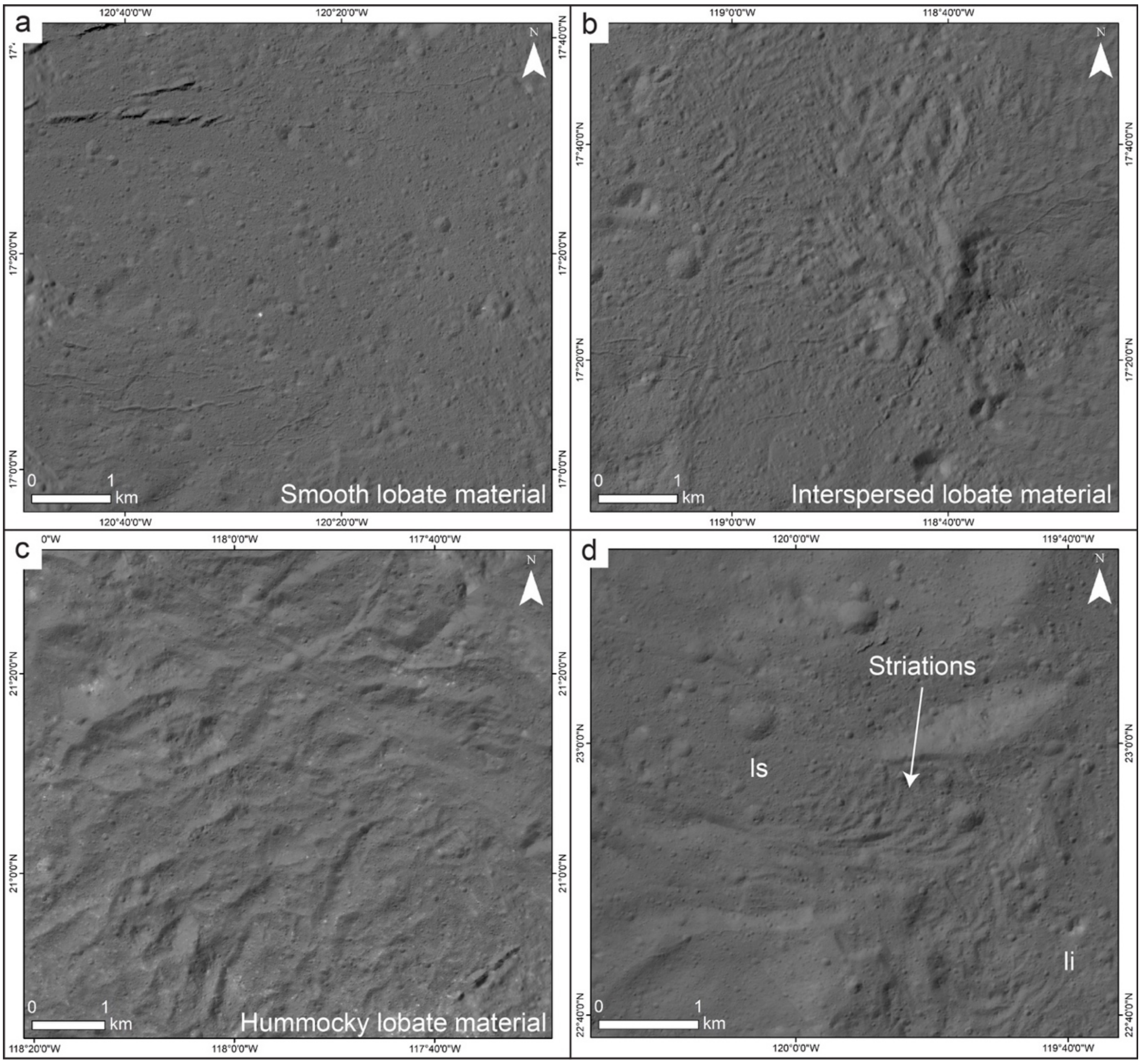

Supplementary Figure 8 Example surface textures of the lobate material. a Smooth lobate material. **b** Interspersed lobate material. **c** Hummocky lobate material. **d** Example of a flow where the end of the smooth lobate material flow (indicated by ls) displayed striations (i.e. the texture of the interspersed lobate material). This flow superposes a region of interspersed lobate material (indicated by li). All images are taken from the base map: the XM2 clear filter FC mosaic[14].

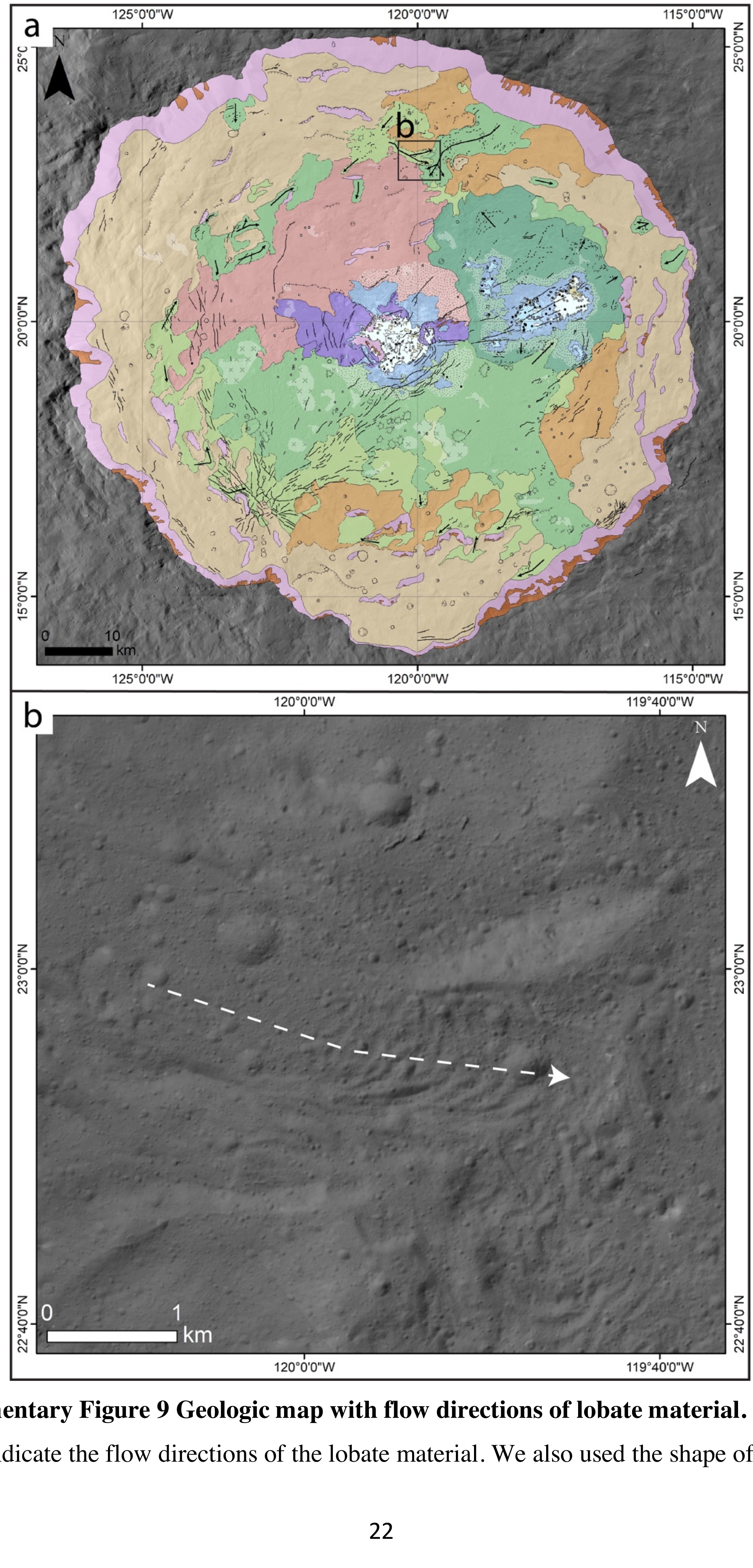

Supplementary Figure 9 Geologic map with flow directions of lobate material. a Black arrows indicate the flow directions of the lobate material. We also used the shape of lobate

flows and the topography to infer flow directions in some locations. The black box indicates the location of image **b**. The legend for the full geologic map is shown in Figure 1a, and the colors and symbols not defined here are the same as in Figure 1a. **b** This image shows an example of when striations were used to infer flow direction (indicated by the white dashed arrow). This image is taken from the base map: the XM2 clear filter FC mosaic[14].

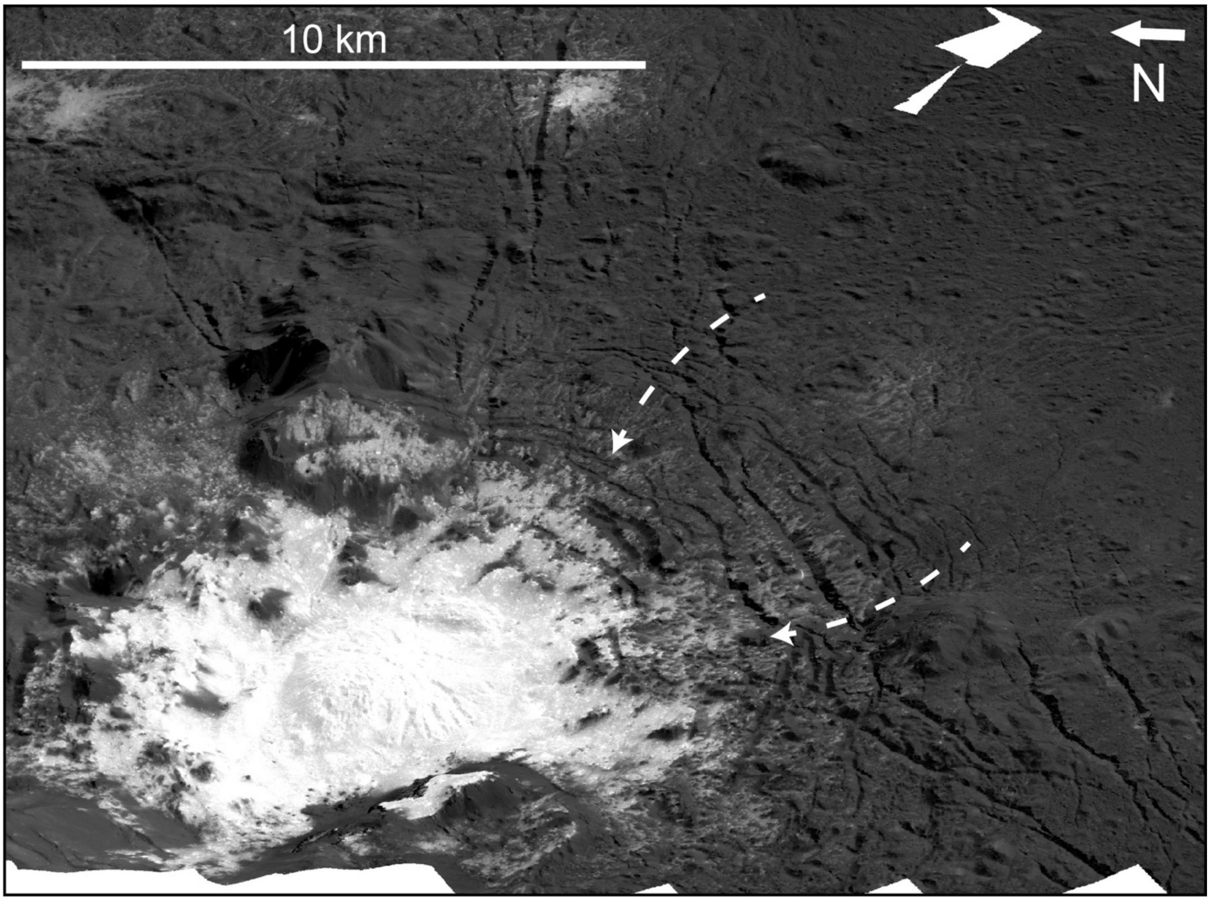

Supplementary Figure 10 Warping of the lobate material. Perspective view of the center of Occator, illustrating how pit formation appears to have warped the northern part of the thick lobate material sheet (indicated by arrows). The ~10 m/pixel XM2 clear filter mosaic has no vertical exaggeration and to make the perspective view we referenced the base mosaic to LAMO DTM[17].

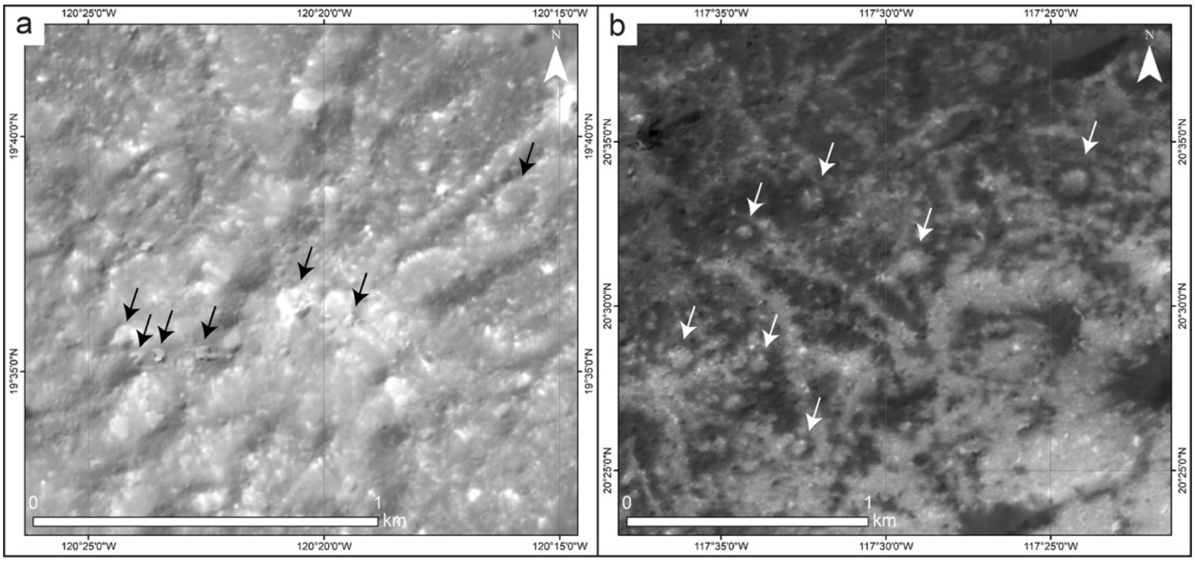

Supplementary Figure 11 The morphology of unusual circular depressions in Occator. a Features that we interpret as possible endogenic pits in Cerealia Facula (indicated by black arrows). **b** Impact craters in Vinalia Faculae that have been partially infilled by bright material (indicated by white arrows). All images are taken from the base map: the XM2 clear filter FC mosaic[14].

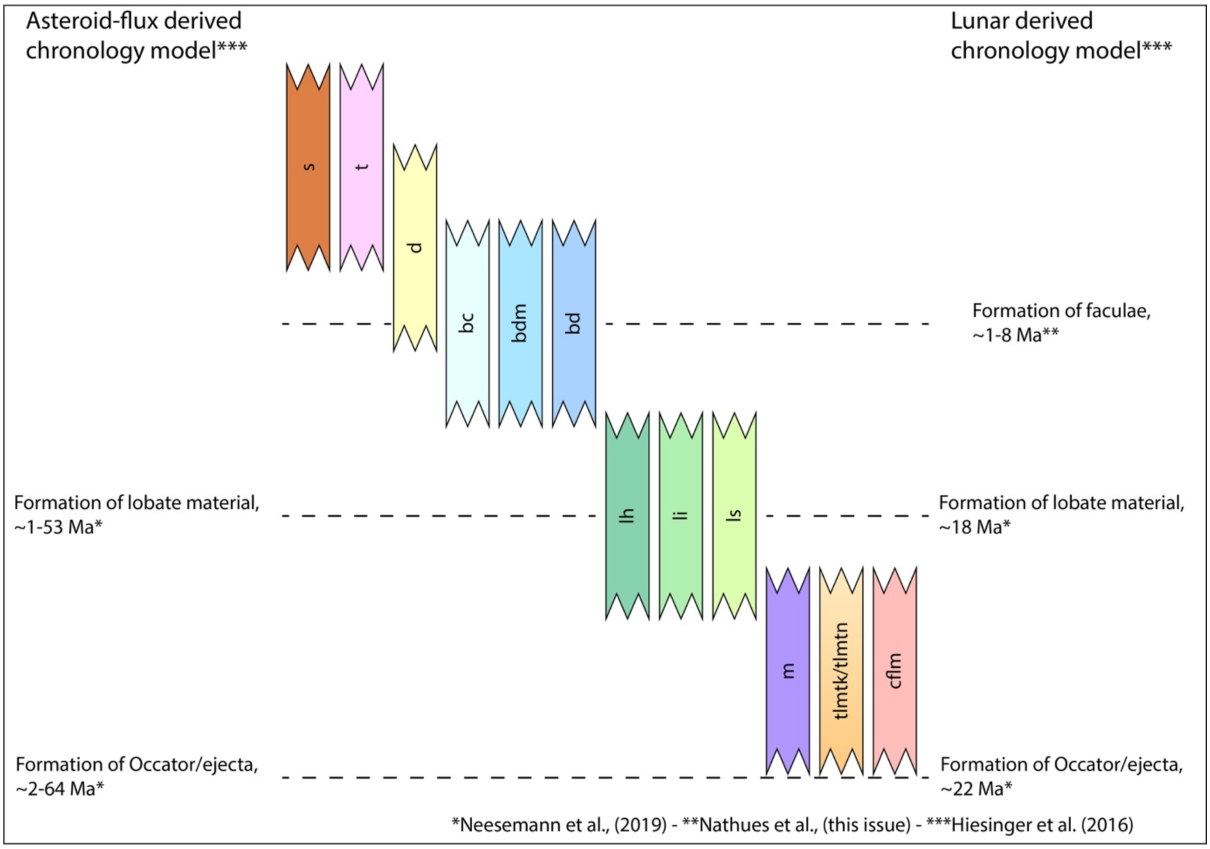

Supplementary Figure 12 Correlation of map units, derived from our geologic map. The relative and absolute ages of our mapped units are illustrated. The differing target properties of Occator's ejecta and lobate material could have contributed to the derived age difference between them. See Methods (subsection, Crater-count-derived model ages) for details about the crater-count-derived model ages. Note that there is a large range in ages obtained from the asteroid-flux derived chronology model because this method incorporates scaling parameters (such as target material strength) that are not tightly constrained[18]. The unit abbreviations are defined in Figure 1a, and the colors and symbols not defined here are the same as in Figure 1a. Neesemann et al. (2019) refers to ref. 18, Nathues et al. (this issue) refers to ref. 19 and Hiesinger et al. (2016) refers to ref. 20.

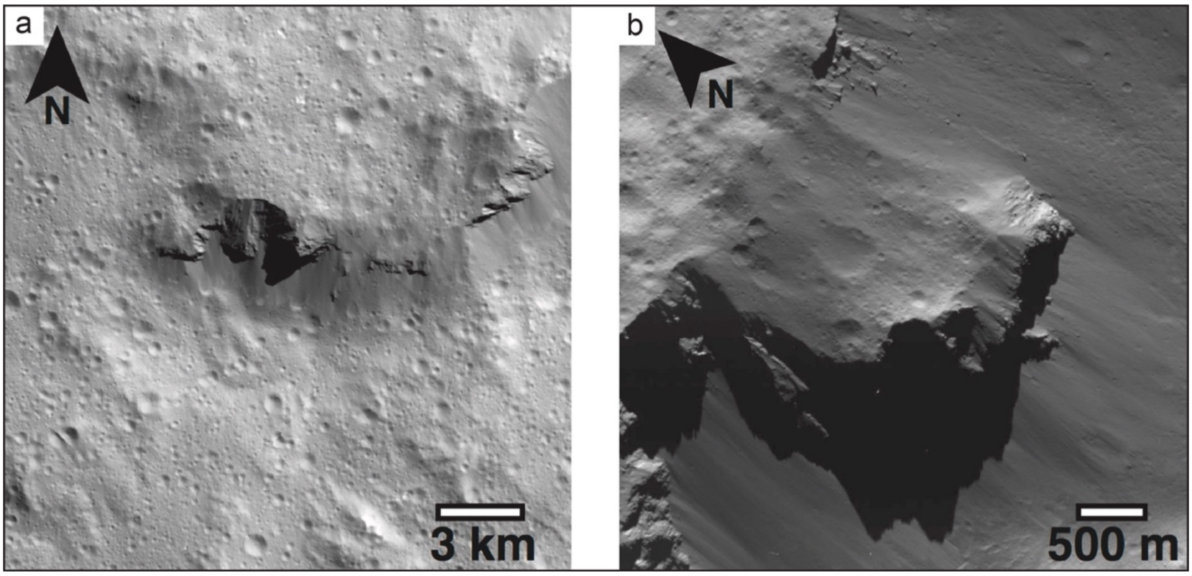

Supplementary Figure 13 Examples of cliffs on crater rims. **a** Steep topography on Urvara crater's northern rim, as seen in XM2 data. **b** A seemingly detached block on Urvara crater's northern rim that may represent a failed cliff, like the features seen in **a**, as seen in XM2 data.
