# Supplementary material for: The varied sources of faculae-forming brines in Ceres’ Occator crater emplaced via hydrothermal brine effusion
Source: Nat Commun. 2020 Aug 10;11:3680. doi: 10.1038/s41467-020-15973-8 (PMC7417532; doi:10.1038/s41467-020-15973-8)
Supplement: Supplementary file 3 — Description of Additional Supplementary Files [file 41467_2020_15973_MOESM3_ESM.docx]

**Description: Supplementary Data 1 - High-resolution JPEG, stand-alone version of the geologic map (Figure 1a).** We include this figure as a separate JPEG file to preserve the high resolution. As in Figure 1a, the geologic map is shown on the basemap at a scale of 1:200,000 and with a simple cylindrical projection. The basemap is shown with no mapping in Supplementary Figure 4.
