# Supplementary figures and images for: The varied sources of faculae-forming brines in Ceres’ Occator crater emplaced via hydrothermal brine effusion
Source: Nat Commun. 2020 Aug 10;11:3680. doi: 10.1038/s41467-020-15973-8 (PMC7417532; doi:10.1038/s41467-020-15973-8)

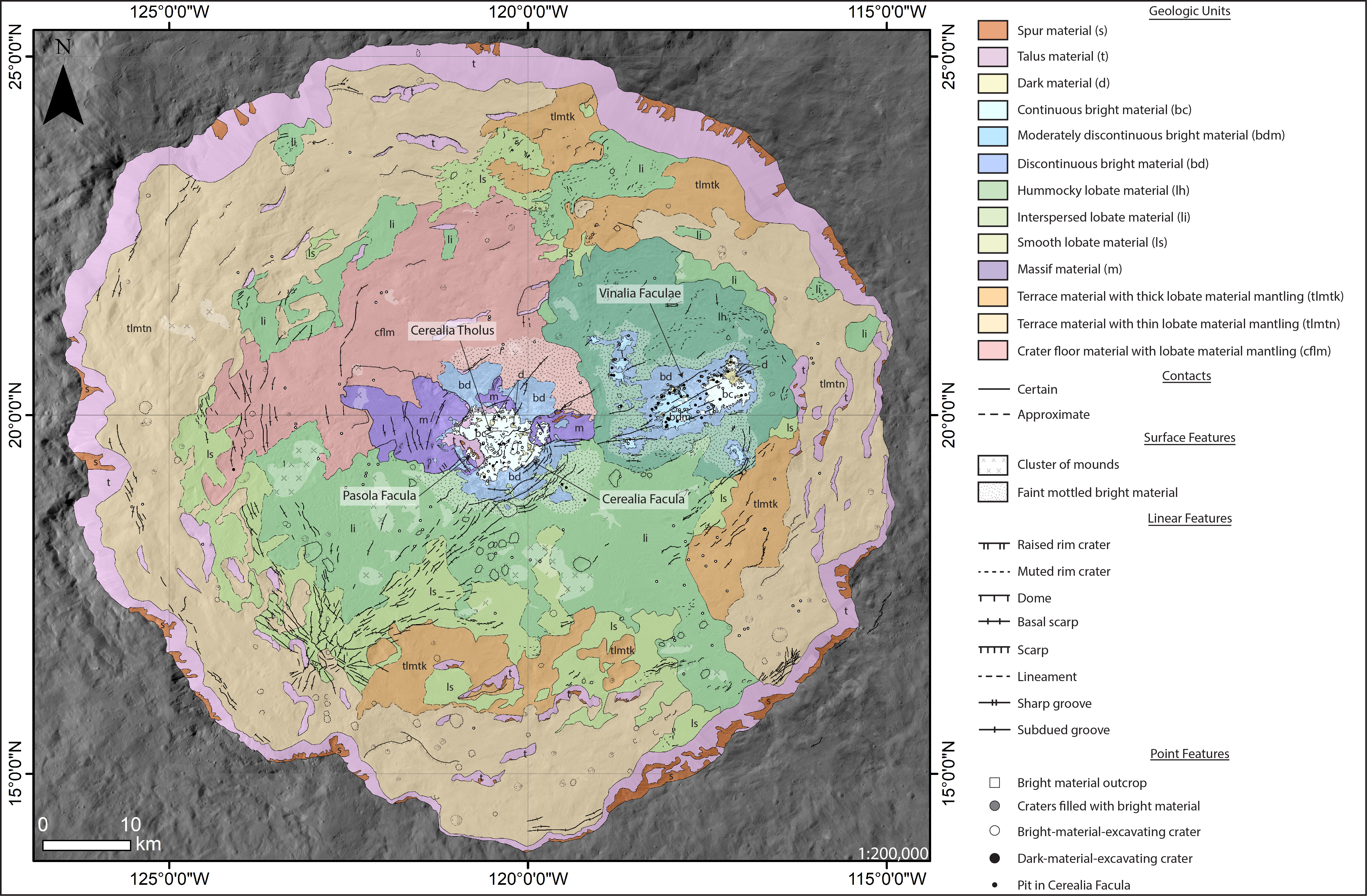

Supplement: Supplementary file 4 — Supplementary Data 1 [file 41467_2020_15973_MOESM4_ESM.jpg]
